# An e-health driven laboratory information system to support HIV treatment in Peru: E-quity for laboratory personnel, health providers and people living with HIV

**DOI:** 10.1186/1472-6947-9-50

**Published:** 2009-12-10

**Authors:** Patricia J García, Javier H Vargas, Patricia Caballero N, Javier Calle V, Angela M Bayer

**Affiliations:** 1 School of Public Health and Administration, Universidad Peruana Cayetano Heredia, Lima, Peru; 2 Instituto Nacional de Salud, Lima, Peru; 3 Department of Microbiology, School of Medicine, Universidad Nacional Mayor de San Marcos, Lima, Peru; 4 Department of Preventive Medicine and Public Health, School of Medicine, Universidad Nacional Mayor de San Marcos, Lima, Peru; 5 Biomedical Research Unit, Asociación Benéfica Proyectos en Informática, Salud, Medicina y Agricultura (AB PRISMA), Lima, Peru; 6 Division of Infectious Diseases, David Geffen School of Medicine, University of California, Los Angeles, Los Angeles, CA, USA

## Abstract

**Background:**

Peru has a concentrated HIV epidemic with an estimated 76,000 people living with HIV (PLHIV). Access to highly active antiretroviral therapy (HAART) expanded between 2004-2006 and the Peruvian National Institute of Health was named by the Ministry of Health as the institution responsible for carrying out testing to monitor the effectiveness of HAART. However, a national public health laboratory information system did not exist. We describe the design and implementation of an e-health driven, web-based laboratory information system - NETLAB - to communicate laboratory results for monitoring HAART to laboratory personnel, health providers and PLHIV.

**Methods:**

We carried out a needs assessment of the existing public health laboratory system, which included the generation and subsequent review of flowcharts of laboratory testing processes to generate better, more efficient streamlined processes, improving them and eliminating duplications. Next, we designed NETLAB as a modular system, integrating key security functions. The system was implemented and evaluated.

**Results:**

The three main components of the NETLAB system, registration, reporting and education, began operating in early 2007. The number of PLHIV with recorded CD4 counts and viral loads increased by 1.5 times, to reach 18,907. Publication of test results with NETLAB took an average of 1 day, compared to a pre-NETLAB average of 60 days. NETLAB reached 2,037 users, including 944 PLHIV and 1,093 health providers, during its first year and a half. The percentage of overall PLHIV and health providers who were aware of NETLAB and had a NETLAB password has also increased substantially.

**Conclusion:**

NETLAB is an effective laboratory management tool since it is directly integrated into the national laboratory system and streamlined existing processes at the local, regional and national levels. The system also represents the best possible source of timely laboratory information for health providers and PLHIV, allowing patients to access their own results and other helpful information about their health, extending the scope of HIV treatment beyond the health facility and providing a model for other countries to follow. The NETLAB system now includes 100 diseases of public health importance for which the Peruvian National Institute of Health and the network of public health laboratories provide testing and results.

## Background

Over 1.9 million people in Latin America and the Caribbean were estimated to be living with HIV in 2007, including 76,000 people in Peru. Peru's HIV epidemic is concentrated among men who have sex with men. While overall adult HIV prevalence was estimated at 0.5 percent [ranging from 0.3%-0.6%], estimates of HIV prevalence among men who have sex ranged from 18 to 22 percent [1]. HIV/AIDS in Peru is urban and primarily impacts men in large cities, especially the capital metropolitan area of Lima/Callao and the jungle region [2]. Youth are also overly-represented in the epidemic; 34 percent of AIDS cases reported to date are among 15-29 year olds [3].

Until 2002, access to highly active antiretroviral therapy (HAART) in Peru was very limited [2]. In 2004, the Ministry of Health initiated universal free access to HAART for people living with HIV (PLHIV) within the framework of the Global Fund to Fight AIDS, Tuberculosis and Malaria and with the participation of civil society and community-based organizations of PLHIV [4].

The HAART program continued widespread implementation during 2005 and 2006. The Peruvian National Institute of Health (INS), which is the Head of the National Network of Public Health Laboratories, was named by the Ministry of Health (MOH) as the institution responsible for carrying out periodic testing to monitor the effectiveness of HAART, consisting of CD4 counts and viral loads [5]. An essential complement to the provision of laboratory testing is a laboratory information system to track each step in the testing process, from the administration of tests to the receipt of test results, thus catalyzing timely decision-making and action around diagnosis, treatment and care [6]. However, at the time that the INS was made responsible for testing, a national public health laboratory information system did not exist in Peru, making the rapid expansion in the quantity and geographic diversity of HAART program clients a sizeable challenge for the INS. Therefore, the NETLAB system was created with the political support of both the INS and MOH.

This article describes key components of the design and functioning of an e-health driven, web-based laboratory information system - NETLAB - created and implemented at the Peruvian National Institute of Health to communicate laboratory results for monitoring HAART. The analysis focuses on the innovative dimension of the system that provides results directly to laboratory personnel, health providers, health administrators and people living with HIV in order to extend the scope of HIV treatment beyond its traditional boundaries.

## Methods

### Needs assessment for the creation of the NETLAB system

The first step in the creation of NETLAB was an in-depth assessment of the public health laboratory system that encompassed the generation of flowcharts in order to determine the step-by-step process for laboratory tests, starting with test-taking at the health facility and ending with the reporting of results to the final user and their publication in the public health system. Once the flowcharts were documented and organized, the NETLAB team shared and discussed them with laboratory personnel. The discussions focused on the identification of critical steps and mechanisms to improve processes and eliminate duplications as well as the determination of the areas of the system that would require systematization.

This paper focuses on the module related to HIV testing, which was developed and piloted as the first component of NETLAB to respond to the new, sizeable demand for monitoring the HAART program. Following the pilot experience with HIV, the NETLAB system expanded to include 100 diseases of public health importance for which the INS and the Network of Public Health Laboratories provide testing and results. This includes dengue, malaria, tuberculosis, yellow fever, measles, German measles, Bartonellosis and Chagas disease.

### Design of the NETLAB laboratory information system

**Figure 1:**
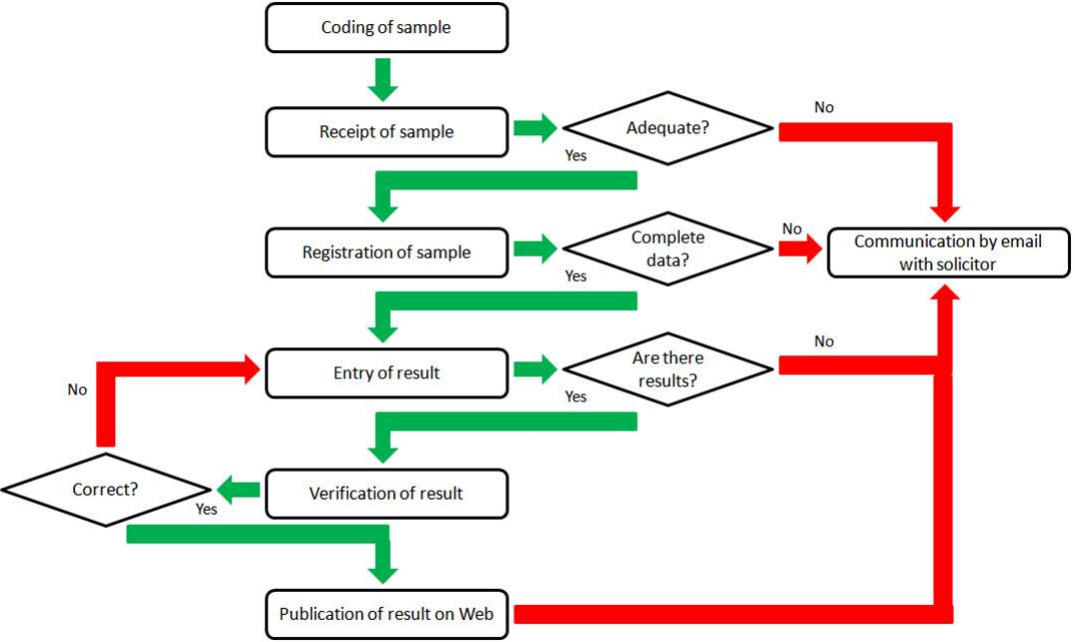
**Flow diagram of process from coding of sample to publication of test result**. Provides a summary of the steps that a sample follows from its reception at the laboratory until the publication of related test results in the NETLAB system.

The discussions between the NETLAB team and laboratory personnel resulted in the preparation and implementation of new flowcharts to visualize the more efficient, better streamlined processes that would become part of the NETLAB system. An example is shown in Figure 1, which summarizes the steps followed by a sample from its reception at the laboratory until the publication of test results in the NETLAB system.

The next step was the design of the NETLAB system itself, which was developed as a modular, integrated system that uses the Windows ^© ^operating system, an MS SQL 2000 ^© ^database manager and applications that use an ASP.NET ^© ^platform. The system has several functions in addition to those that are described in detail below, including an on-line catalog of all laboratory tests offered and their respective processing times.

Since the NETLAB system targeted different user groups who accessed the system from different locations, assuring confidentiality was a key focus. NETLAB complies with the Peruvian General Health Law, Article 120, which states that health information generated by public entities that could affect personal or family privacy or personal image is not subject to the Law that makes such information part of the public domain. The system also respects Law 26626, which specifies that HIV results are confidential. NETLAB complies with this legislation by encrypting users' usernames and passwords in the system database and by including a digital certificate of the authenticity and integrity of the information transferred. Peruvian law specifies that digital certificates have the same legal validity as hand-written signatures.

### Evaluation of the NETLAB system

Two evaluations of the system were carried out during the first year and a half of NETLAB's operation. The first evaluation applied quantitative surveys in a one-on-one interview format with health providers who administer HAART, laboratory personnel and PLHIV in Lima/Callao and 22 of the 23 other regions of Peru five to six months following the initial implementation of NETLAB [7]. The second evaluation included a quantitative survey applied as an interview with the same populations and two focus groups with PLHIV in Lima/Callao and in 4 other regions (Arequipa, Ica, Lambayeque and Loreto) and was carried out 14 to 15 months post initial implementation of the system. The evaluations included all laboratory personnel, physicians, nurses and midwives from the HAART teams at each health establishment who were present at the establishment on the day of survey application. They also included all PLHIV who were on HAART or had at least 2 CD4 counts and viral loads and who were present during survey application at the respective sites. In the case of a high flow of PLHIV at a given establishment on the day of the visit, every third PLHIV was invited to participate. All PLHIV gave their verbal consent to participate in the evaluation.

## Results

### Basic components of the NETLAB system

The NETLAB system was developed during the last quarter of 2006 and was implemented and started collecting information on January 30, 2007. It has three main components: (1) a registration component for entering the data from the moment a sample is received until test results are complete; (2) a reporting component to communicate the test result to different users; and (3) an educational component.

The ***registration component of NETLAB ***gathers two types of information: one about the sample and one about the patient. Registration starts with the assignment of a barcode to each sample when it reaches the reception area of the laboratory and the use of the same barcode throughout the analysis and reporting process. The use of the barcode minimizes human error and also enables sample tracking. Basic information about the patient is also recorded using a unique identification code for each person. This number corresponds to the National Identification Document (DNI) that all Peruvians receive and keep until death. This number, which used to be assigned at age 18, is now given starting at birth. The DNI number allows the NETLAB system to maintain patient-specific information and search for historical information about patients in the database.

The NETLAB system also has the capacity to automatically detect duplicate or omitted patient data and request verification from the individual or establishment that sent the sample. Email messages are sent to the health provider, health establishment or laboratory that provided the sample in order to seek further information about the client and sample and if necessary, request a new sample, thus improving the quality of test results and information about each patient.

The system allows the registration of test results both manually and automatically, all of which are double-checked prior to publishing. Automatic registration is available for CD4 counts since the cytometer is connected to the NETLAB system database through a platform. Before any results are made accessible to the public, they are verified by the laboratory coordinator.

There is one additional function that has been very useful for follow-up of samples. This function is based on a module that marks pending results with green, yellow and red flags (like a traffic light) if the sample processing is on time (according to the on-line catalog times), delayed by one day after the expected time for results, or delayed two or more days longer than expected. This is quite helpful for monitoring laboratory performance and the quality of services provided by the laboratories and for improving patient care, since delayed results and health care decisions could jeopardize patients' treatment and health.

**Figure 2:**
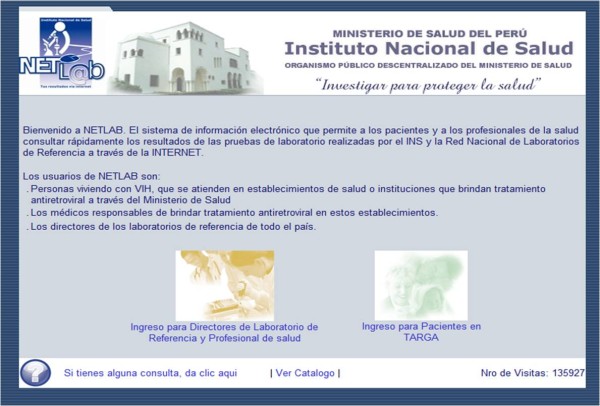
**Main NETLAB screen**. Shows the primary NETLAB screen, including points of access for laboratory personnel, health providers and health administrators (left side) and for HAART patients (right side).

**Figure 3:**
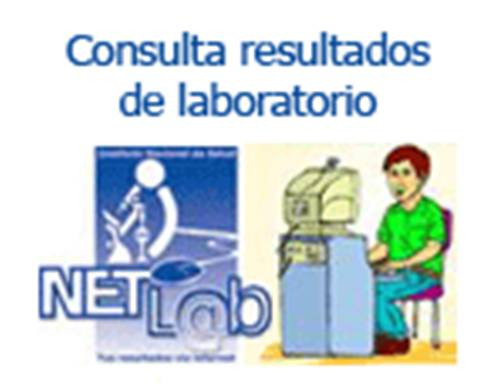
**NETLAB access icon**. Shows the NETLAB access icon, which is displayed on the main webpage of the Peruvian National Institute of Health and provides direct access to the main NETLAB screen shown in Figure 2.

The ***reporting component of NETLAB ***makes the system accessible to all users over the Internet (Figures 2 and 3), including an expanded number of laboratory personnel, health providers, health administrators and PLHIV. The system assigns a unique profile to each group of users based on the type of information access that each group requires either as a human right, as is the case with PLHIV, or in order to carry out their professional responsibilities, as is the case with the other groups. Additionally, access to this information will further engage health service users in their own treatment and care and should also help them to become more adherent to treatment. Through NETLAB each PLHIV can access all of his or her test results carried out through the Ministry of Health, independent of the health establishment where the test was taken, and health providers in the HAART program can access the historical results of all of her or his HAART patients. Each individual user accesses the NETLAB system through a unique username and password that is known only to the individual. Users can enter the system through any computer that has Internet access, either at a health establishment, a public Internet cafe or at home.

**Figure 4:**
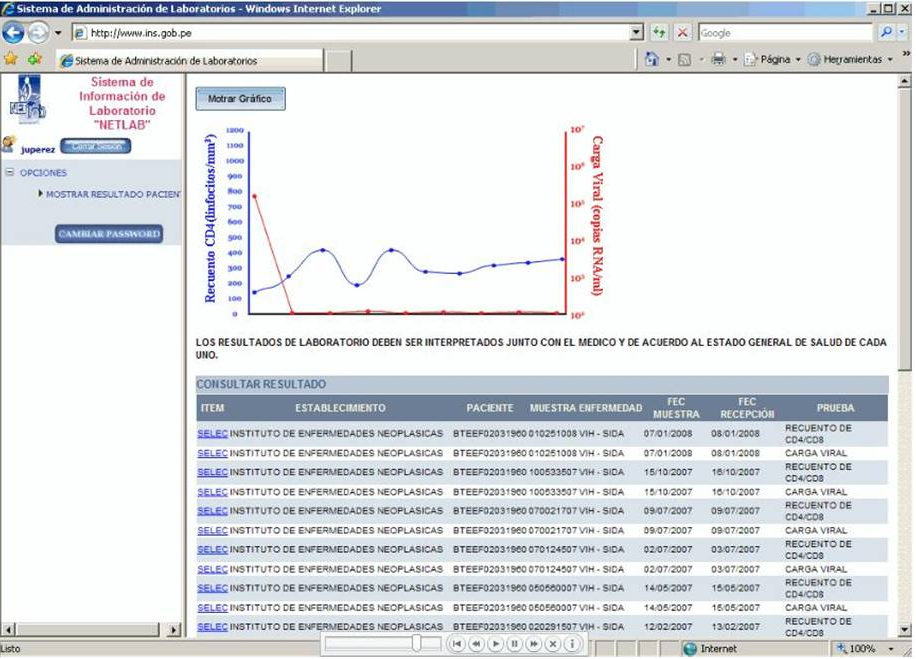
**Presentation of individual CD4 count and viral load results over time**. Shows historical test results for a HAART patient, including 10 CD4 counts and 10 viral loads, which are available to the patient and the patient's health provider through the NETLAB system.

NETLAB results are provided in a user-friendly, easy-to-understand format and users are able to access both current and historic test results (Figure 4). Simple text is used to show the type of test, the establishment where it was taken, and the numeric result for all of the PLHIV's HIV-related tests. Simple graphs are also presented, showing the individual PLHIV's test results over time. In addition to obtaining test results, NETLAB users are able to provide constant feedback and report any problems or questions. The main NETLAB screen prominently displays a feedback form through which the user can describe difficulties and questions and send a message directly to NETLAB staff, who respond within 24 hours.

**Figure 5:**
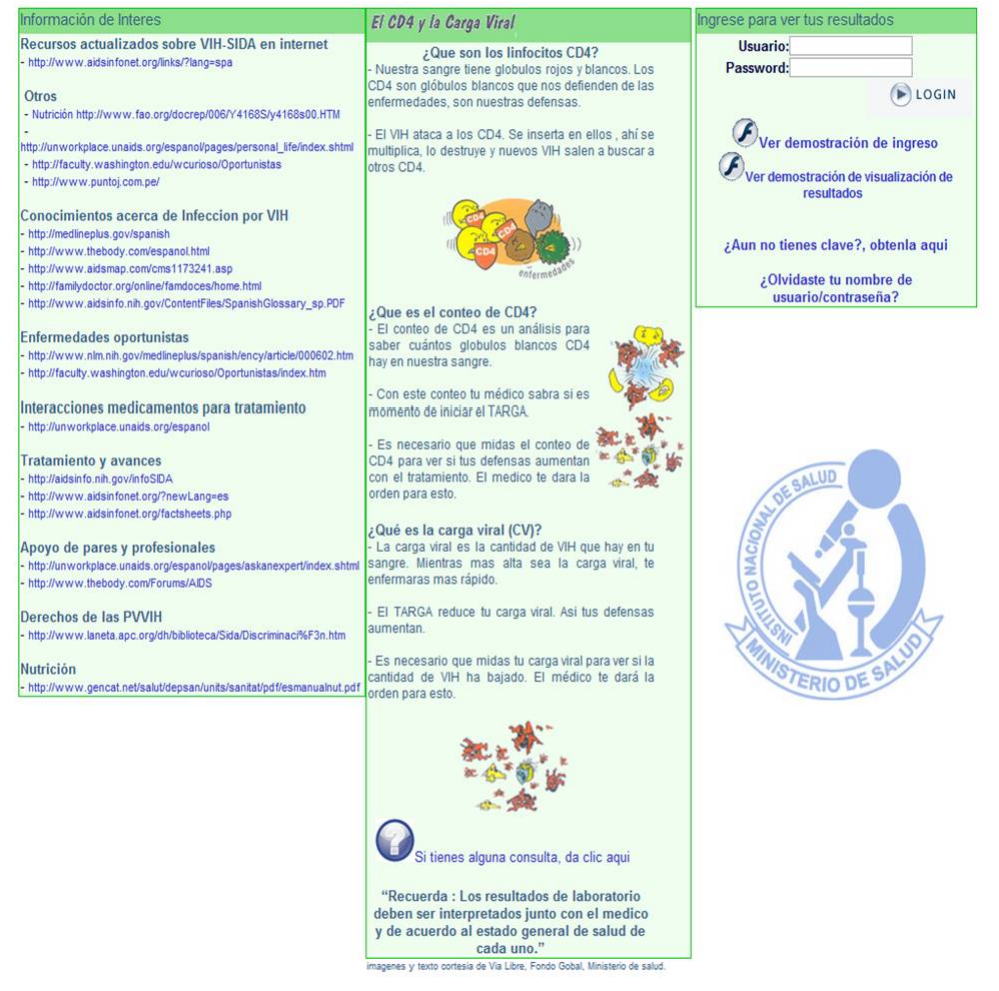
**NETLAB educational material for people living with HIV**. Shows the NETLAB screen of educational information for PLHIV, which includes an explanation of CD4 cells, CD4 counts and viral loads, as well as links to webpages that might be of interest to PLHIV.

The ***educational component of the NETLAB ***system significantly expands its utility for all users. On the main user screens, prior to entering the username and password, all users and others who might be interested are able to access educational information. The screen for PLHIV explains CD4 cells, CD4 counts and viral loads in a simple-text format that includes graphics (Figure 5). The screen also provides numerous links to high-quality, user-friendly webpages that might be of interest to PLHIV, about HIV/AIDS, opportunistic infections, treatment and advances, support, nutrition and PLHIV rights. Also, all results provided to PLHIV are accompanied by a message that encourages the user to discuss her or his test results with the health provider. The screen for health providers and administrators provides links to updated information about HIV/AIDS, resources on related clinical care and research, and scientific journals.

In Table 1 we present a summary comparison of the way results were offered through the INS before NETLAB and with the NETLAB system.

**Table 1:** Characteristics of the management of test results before NETLAB and with NETLAB

Characteristic *(based on Eysenbach's 10 e's)*	Before NETLAB	NETLAB
**Efficiency** **(e #1)**	The records showed an average of 6,516 CD4 counts and 6,744 viral loads processed per year between 2004 and 2006.	The NETLAB system recorded, processed and registered 16,053 CD4 counts and 16,047 viral loads in 2007.
	After the test was completed, paper-based laboratory results took an average of 60 days [range 15-120 days] to reach a group of selected users.	After the test is completed, Internet-based results are available immediately to the appropriate users. The average time to publication of results is 1 day [range 1-22 days].
**Enhanced quality** **(e #2)**	No system existed to track samples throughout the country.	NETLAB uses the same barcode system throughout the country, minimizing human error and enabling sample tracking.
	No system existed to check the quality of samples and verify information or request new samples.	NETLAB checks all samples for quality and readability, and requests additional information and/or new samples from health providers and establishments through email messages.
	The laboratory results were manually entered and had no function to detect duplication or omission of patient data.	NETLAB automatically detects the duplication or omission of patient data and verifies the information through automatic email messages.
	There was no mechanism to verify the test results entered in the database.	The laboratory coordinator verifies all test results before allowing their publication in the NETLAB system.
**Evidence-based** **(e #3)**	No periodic evaluation of the system was carried out.	The "traffic light" system provides real-time monitoring of the system. Additionally, repeated evaluations of NETLAB have been carried out since its inception.
**Enable information exchange (e #4) and Extend healthcare beyond its traditional boundaries (e #9)**	Laboratory results were not accessible for PLHIV and only for a small number of users.	Laboratory results can be accessed by PLHIV or by health providers, depending on the individual's user profile.
	Results were available in paper format and sent through regular mail, which could take months to become available.	Results are available on the Internet almost in real time as soon they are ready. They're presented in a user-friendly format and include current and historical test results in text and graphic format.
	There was a limited number of users (66 total).	NETLAB has a high number of users (2,037 total), which continues increasing.
**Empowerment of clients (e #5), Encouragement of new relationship between providers and clients (e #6), and Equity (e #8)** **Education of clients and providers** **(e #7)**	There was no access for people living with HIV and limited access for health providers.	There are currently 944 people living with HIV and 1,093 health providers who have access to NETLAB.
	There was no mechanism for client feedback.	There is a user feedback function on the NETLAB page. All comments are responded to within 24 hours.
	No HIV-centered education was provided.	User-friendly, user-group specific information is provided to NETLAB users and others who access the webpage.
**Ethics** **(e #10)**	Results in the database were only available to laboratory personnel. In order to have legal value, all results needed to be printed and signed and then sent to the health establishments, which could take months.	Results are available at any time on the Internet and confidentiality is maintained through a system of usernames and passwords.
		The digital certificate means that all NETLAB results have legal value whether they are viewed, downloaded or printed.

### Achievements of the NETLAB system

In 2007, tests for diagnosing and monitoring HIV/AIDS represented 41.2 percent of the total production of the Peruvian National Institute of Health (INS). In Peru, CD4 counts are monitored at baseline in recently-diagnosed patients and then periodically, at least once per year, both for PLHIV who are currently taking HAART and for those who do not yet take HAART. Samples for CD4 counts are taken at health establishments throughout the country and then sent to the INS or one of ten regional laboratories for analysis. Viral loads for PLHIV are also taken at least once a year and sent to the INS for analysis.

The NETLAB system processed and registered 16,053 CD4 counts and 16,047 viral loads in 2007, a significant increase over an average of 6,516 CD4 counts and 6,744 viral loads per year between 2004 and 2006. The number of PLHIV with recorded CD4 counts and viral loads also increased, from 11,169 in early 2007 to 14,667 at the end of 2007 and 18,907 as of September 2008. NETLAB has greatly improved the efficiency of the process for reporting test results. Before NETLAB, laboratory results were paper-based and could take an average of 60 days [range 15-120 days] to reach the health provider. Through the NETLAB system, laboratory results are available on the NETLAB webpage to all users who should have access on the same day that results are ready. The average time from completion of testing to publication of results has decreased to 1 day [range 1-22 days].

The number of individuals who are able to access results has expanded significantly, from 66 users prior to the implementation of NETLAB in late-2006 to 2,037 users through NETLAB as of mid-2008. Current NETLAB users include 944 PLHIV and 1,093 health providers.

### Evaluation and improvements in the NETLAB system

Since the start of NETLAB, two survey-driven evaluations were carried out with PLHIV and health providers, all of whom are involved in the HAART program but not necessarily in NETLAB. The first evaluation took place 5-6 months post NETLAB initiation and the second took place 14-15 months after NETLAB began operating.

#### People living with HIV (PLHIV)

As shown in Table 2, over half of the PLHIV interviewed in both surveys reported knowing how to use the Internet and a high proportion of interviewees used the Internet at least once a week. The most remarkable changes were seen in the awareness of NETLAB, which increased from 9.5 percent to 52.3 percent between the first and second surveys. Some questions about exposure to training on the use of NETLAB and possession of a NETLAB password were only asked in the second survey. Around 16 percent reported having received training on NETLAB use, 11 percent reported already having a password and 14 percent reported having requested a NETLAB password (data not shown in table).

**Table 2:** Internet use and awareness of NETLAB among people living with HIV and health providers

	5-6 months post initiation of NETLAB	14-15 months post initiation of NETLAB
**PLHIV**	**N = 201**	**N = 457**

	**% (n)**	**% (n)**

Age	32.9 (avg.)	35.1 (avg.)

Male biological sex	67.2 (135)	60.2 (275)

Knows how to use Internet	50.7 (102)	56.0 (256)

Has heard of NETLAB	9.5 (19)	52.3 (134)

		

**Health Providers**	**N = 246**	**N = 85**

	% (n)	% (n)

Knows how to use Internet	95.5 (235)	100.0 (85)

Uses Internet 2 or more times per week	99.6 (234)	94.1 (80)

Has heard of NETLAB	50.4 (124)	96.5 (82)

Has a NETLAB password	35.4 (87)	76.8 (63)

*Health providers includes professionals who work in the HAART program at different health establishments, including doctors, nurses and midwives, and laboratory personnel.

During the later evaluation, 14-15 months post the start of NETLAB, twenty PLHIV who had experience with NETLAB also participated in focus groups. Participants made numerous recommendations, including the importance of making the link to the NETLAB system more accessible and visible and making the process for obtaining a username and password less bureaucratic and available on-line. All of their recommendations were acted on within a three-month period, demonstrating NETLAB's commitment to the population it serves. The NETLAB icon was moved to a prominent position on the INS web page and the process for gaining access to the creation of a username and password was changed from a paper-based to an on-line system, which still maintains strict ethical guidelines. The capacity to generate a new username and password in the case of a lost or forgotten username or password has also been included on-line. In terms of the NETLAB system itself, participants recommended the addition of educational information that is brief, with minimal text and in simple language. These recommendations were also integrated into the system and are now part of the educational component, described above.

#### Health providers

Internet use was also very high among health providers, with 95.5 percent of respondents during the first survey and all respondents during the second survey reporting knowledge of how to use the Internet and almost everyone accessing the Internet at least two times a week. As with PLHIV, there was an increase in awareness of NETLAB among providers between the first and second surveys, with awareness nearly doubling from 50.4 percent to 96.5 percent of providers. The proportion of providers who had a NETLAB password more than doubled, from 35.4 percent to 76.8 percent. Of the 82 health providers who had heard about NETLAB in the second survey, 68 or 82.9 percent of health providers had received training in how to use NETLAB. All laboratory personnel had received training, compared to 8 in 10 non-laboratory health providers (80.7%) (data not shown in table).

## Discussion

Around the globe, there are limited examples of information systems to support HIV treatment in developing countries. National systems are few and examples in the Latin America and Caribbean (LAC) region include Brazil [8] and Cuba [9]. Other systems in the LAC region are limited to a small number of health establishments, as seen in the case of the Partners in Health-Zanmi Lasante system in Haiti [10]. In other regions, a model example is the AMPATH medical record system for HIV/AIDS care that has now been expanded to 19 health centers in Kenya [11] and is based on OpenMRS, the open-source Medical Record System [12]. Other examples of electronic medical databases to track antiretroviral therapy in Africa, South America and Asia were also reviewed in a recent article [13]. NETLAB represents an important contribution to information systems to support HIV treatment given its integration of all of the components of e-health and its nationwide reach while also building local capacity for all system users, including health providers and people living with HIV.

"E-health" takes a step beyond the creation of an information system by delivering services and information through the Internet and related technologies and by integrating other innovations. Eysenbach (2001) recently proposed 10 "e's" that should form part of "e-health." Some of these "e's" are already part of a health information system, including 1) **e**fficiency, 2) **e**nhanced quality, 3) **e**vidence-based, and 4) **e**nabling information exchange and communication. The remaining "e's," however, represent an additional step toward making health information as beneficial as possible for all involved actors: 5) **e**mpowerment of clients; 6) **e**ncouragement of a new relationship between clients and health providers; 7) **e**ducation of clients and providers; 8) increased **e**quity; and 9) **e**xtension of health care beyond its traditional boundaries. These steps all need to consider the final "e," 10) **e**thics [14].

NETLAB meets all of the dimensions of e-health as defined by Eysenbach (2001). NETLAB contributes to improved quality and efficiency (criteria 1-2) by tracking samples, by checking samples for quality and readability, by verifying the test results entered in the system, by checking the system for patient duplication and omission, and by shortening the reporting time through the use of a Web-based instead of a paper-based system. The NETLAB system also takes additional steps toward achieving the full definition of e-health by enabling information exchange through the communication of information (criteria 4) through user-friendly text and graphics to a wide range of populations including PLHIV. The communication of informative, understandable data to all populations involved in HAART affirms NETLAB's commitment to equity (criteria 8) and the direct inclusion of PLHIV as NETLAB users demonstrates the system's dedication to empowering PLHIV and encouraging a new relationship between PLHIV and their health providers (criteria 5-6), thus moving health care beyond its traditional boundaries (criteria 9). Further empowerment for all NETLAB users is possible through the user feedback function on the NETLAB screen and through the educational information that is provided for different user groups (criteria 7). All of these groups have been involved throughout the design and implementation of NETLAB and their feedback is integrated directly and rapidly into the system, demonstrating NETLAB's commitment to an additional e-health criteria: evidence-based systems (criteria 3). Finally, the system ensures strong ethics by including a digital certificate with all results (criteria 10).

This consistent user feedback has translated into increased awareness and use of NETLAB among PLHIV and health providers, as demonstrated in the difference between the earlier and later evaluation results. These results also show, however, that more work needs to be done in terms of ensuring that PLHIV have Internet access and that they are aware of NETLAB and using the system if they choose to do so. Numbers thus far are consistent: of the approximately 8,000 PLHIV on HAART in Peru, 944 of them are NETLAB users. This signifies that over 10 percent have gained access to the system in only a year and a half and with few NETLAB enrollment outreach activities. NETLAB personnel are currently working directly with support groups for PLHIV, which are active throughout the country, in order to expand access to the Internet and awareness of and access to NETLAB. Efforts are also underway to reach the target of universal knowledge and access for health providers, with a focus on non-laboratory providers. Trainings for all potential user groups include information on how to access the NETLAB system, on the system itself, and on the system's different benefits for providers, including individualized test results and supplemental education. All training sessions also offer additional opportunities for feedback.

## Conclusion

It is important to close by underscoring that the NETLAB laboratory information system is directly integrated into the National Network of Public Health Laboratories and that it works to further streamline existing processes at the local, regional and national levels while expanding access to key information for a broader range of users. NETLAB was honored with a governmental best practice award in 2008, in the category of citizen services http://www.ciudadanosaldia.org/premiobpg2008/Resultados/default.htm. NETLAB will continue to transform and change based on user feedback in order to serve as the best possible laboratory management tool at all levels of implementation and as the best possible source of timely laboratory information for health providers and people living with HIV, with the aim of further extending the scope of HIV treatment in Peru and providing a model for other countries to follow. Sustainability of such a system is a critical issue and needs to address how to maintain the continuity of this and other important health information systems when changes in institutional leadership take place. Initial sustainability is apparent in the current state of NETLAB: the 24 regional laboratories and the main regional hospitals are all connected to the system. The future goal is to expand to smaller health centers and health posts and to have more users from the different user groups.

## Competing interests

The authors declare that they have no competing interests.

## Authors' contributions

PJG conceived of the study and, together with JVH, participated in the development and implementation of NETLAB and carried out earlier analyses and evaluations of the system. PCN participated in the implementation and evaluation of the system. JCV designed the system. AMB drafted the manuscript. All authors revised and approved the final manuscript.

## Funding Statement

Angela Bayer is currently a Postdoctoral Scholar under NIH NIMH grant T32MH080634-03.

## Pre-publication history

The pre-publication history for this paper can be accessed here:

http://www.biomedcentral.com/1472-6947/9/50/prepub
